# *FXD-CSD-GUI*: a graphical user interface for the X-ray-diffraction-based determination of crystallite size distributions

**DOI:** 10.1107/S1600576719012159

**Published:** 2019-10-22

**Authors:** Sigmund H. Neher, Helmut Klein, Werner F. Kuhs

**Affiliations:** a GZG Crystallography, University of Göttingen, Goldschmidtstrasse 1, Göttingen, 37077, Germany

**Keywords:** FXD-CSD, X-ray diffraction, Bragg intensities, crystal size distribution, two-dimensional detectors

## Abstract

Bragg intensities can be used to analyse crystal size distributions in a method called FXD-CSD, which is based on the fast measurement of many Bragg spots using two-dimensional detectors. This work presents the Python-based software and its graphical user interface *FXD-CSD-GUI*. The GUI enables user-friendly data handling and processing and provides both graphical and numerical crystal size distribution results.

## Abstract 2

A graphical interface to the Python module *fxdcsd*, which analyses crystallite size distributions via Bragg intensities measured with two-dimensional detectors, is described.

## 1. FXD-CSD method   

Fast X-ray diffraction crystal size distribution analysis, FXD-CSD (Neher *et al.*, 2018 ▸), is a reference-material-based X-ray diffraction method to derive volume-based CSDs for polycrystalline materials and powders. FXD-CSD uses the ratio of the integrated diffraction intensity of a reference to its irradiated crystal volume as a scaling factor (S1); the sample and reference do not need to be composed of the same substance. With this scaling factor, the volume CSD of an unknown sample is deduced. To obtain the integrated intensities of the sample and reference, stepwise rotation diffraction measurements have to be carried out for both. These measurements are performed with a diffractometer equipped with an area detector. To carry out the subsequent FXD-CSD analysis, the crystallographic structures (the structure factors and unit-cell volume) of the sample and reference have to be known. For intensity scaling, the CSD information of the measured reference material should be available, *e.g.* as measured with scanning electron microscopy and extracted by image analysis. If no full CSD is available, the mean value can be used instead as a point of reference.

Stepwise rotation measurements, as used in FXD-CSD, produce large data sets in a short time. These data are reduced by extracting the individual integrated intensities of the Bragg spots detected. All extracted intensities are corrected for geometric effects (*i.e.* Lorentz correction) and scaled with the scattering power of the corresponding *hkl* plane (*i.e.* the structure factor *F* ^2^_*hkl*_). This data reduction and scaling process is quite time intensive and therefore can only be carried out routinely with custom-built software, the Python module *fxdcsd*. Its graphical user interface, *FXD-CSD-GUI*, is presented in the following.

## 2. Graphical user interface: *FXD-CSD-GUI*   

To facilitate the use of the *fxdcsd* Python module for uninitiated users, a graphical user interface, *FXD-CSD-GUI*, is provided and made available as part of the *fxdcsd* Python module. *FXD-CSD-GUI* allows the user to perform all necessary steps for a complete FXD-CSD analysis. Here we summarize the tools and information needed before an FXD-CSD analysis can be undertaken; for more details, we refer to Neher *et al.* (2018 ▸) and the user manual available at https://owncloud.gwdg.de/index.php/s/JGluHlEp0pEUcAH and as supporting information for this article. The following items are needed to perform a CSD analysis:

(i) A diffractometer with a two-dimensional detector and at least one sample rotation axis.

(ii) A reference powder with known CSD in the lower µm range (∼0.5–100 µm). The CSD should ideally be available as an individual crystal volume information data set. At least the characteristic numerical values (*e.g.* mean value and distribution spread from a histogram fit) of the CSD histogram are needed.

(iii) Stepwise rotation measurement data sets (consecutive frames, *e.g.* Bruker .sfrm or .tif files) from the reference and sample acquired under the same conditions.

(iv) The crystal structure of the reference material and the sample material to be investigated. This information is needed to calculate the structure factors and the cell volumes, which are necessary for a correct intensity scaling. The structure factor and cell volume calculations are not carried out using *FXD-CSD-GUI*. For this purpose we suggest, for example, * PowderCell* (Kraus & Nolze, 1996 ▸) or *Mercury* (Macrae *et al.*, 2008 ▸).

**Figure 1:**
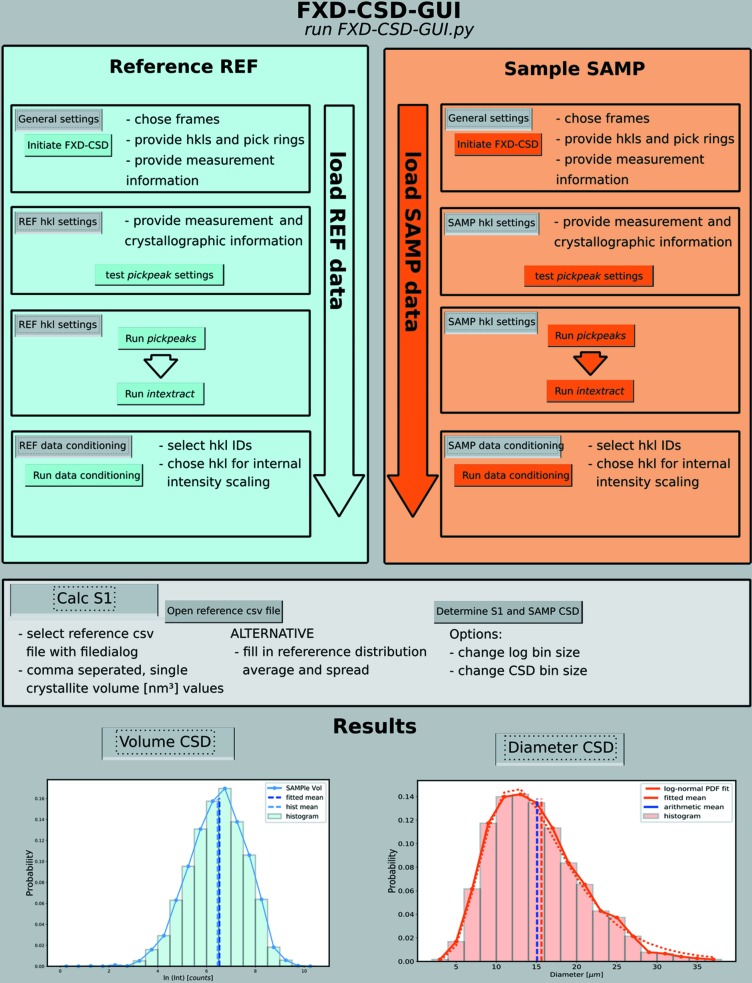
*FXD-CSD-GUI* flow chart.

During the analysis with *FXD-CSD-GUI*, the following steps are performed; the flow diagram in Fig. 1 ▸ illustrates these steps, and details of the software tasks are provided by Neher *et al.* (2018 ▸):

(i) Initiating the analysis by loading the detector frames and picking the *hkl* rings that are to be analysed (carried out for the reference material and sample). For this task, we recommend having a plotted and indexed powder pattern at hand. Reference and sample are abbreviated as REF and SAMP within the GUI.

(ii) Providing the required structure factor and unit-cell size information for both the reference and the sample and for each ring.

(iii) Testing and final application of the *pickpeaks* function. In this step, the program detects the diffraction peaks by an intensity threshold operation. The threshold defines the intensity value used to separate the background intensity from the diffraction spot intensity. The threshold intensity level is set by the user. During testing, each parameter can be tuned manually. Most important here is the already mentioned threshold and the ring_width parameter, which is the width of the ring-shaped area of interest. Running the *pickpeaks* function can take, depending on frame size and frame number as well as the computer power, up to several hours.

(iv) Running the *intextract* function, which first allocates the diffraction spots detected in adjacent frames and then integrates the diffraction spot intensities.

(v) Conditioning the REF and SAMP data. The integrated intensities are corrected for the Lorentz–polarization factor and are scaled with their structure factors with respect to one user-chosen *hkl* ring. The ratio between the structure factors, normalized to their unit-cell volumes, of the two chosen *hkl* rings (reference and sample) is called the S2 scaling factor [see Neher *et al.* (2018 ▸), Section 3.1]. All parameters can be manipulated by the user and one can choose which *hkl* rings will be included in the final CSD analysis. The results are shown as intensity histograms.

(vi) Determining the S1 scaling factor between the mean of the measured reference intensity histogram and the mean value of its volume distribution – measured independently [*e.g.* via light scattering or scanning electron microscopy; see Neher *et al.* (2018 ▸), Section 4]. The used mean values are determined via automated distribution fitting. The user can choose between a lognormal, Gaussian or skewed-Gaussian model. In addition, it is possible to add a custom model (see the user manual). The volume distribution either can be loaded as a csv file with comma-separated single-crystallite volume (nm^3^) values or can be provided as numerical values (*e.g.* average crystallite size and distribution spread). In the case of numerical values, the input is used for plotting a log­normal distribution (see the user manual). In our experience, a lognormal distribution is the most likely distribution shape when the reference material is produced via mechanical crushing of larger single crystals and crystal size fractions are separated from it [see Neher *et al.* (2018 ▸), Section 4 and Section S4 of the supporting information].

During the analysis, all necessary numerical inputs are explained within the interface and highlighted according to their state of input, *e.g.* mandatory input parameters are highlighted red when missing and turn green when they are provided consistently. The results are provided numerically and are shown graphically. The graphics can be exported in various formats (.png, .jpeg, .svg or .pdf).

## 3. Software and hardware implementation   

The program has been tested on 64 bit Windows (7 and 10) and Ubuntu Linux (16.4) machines but should also run on macOS. It is written in Python 2.7 using the default *Tkinter* (Lundh, 2005 ▸) module for GUI programming. The plotting and frame visualization are done with the *Matplotlib* (Hunter, 2007 ▸) module. Reading the diffractometer frames is done with *fabIO* (Knudsen *et al.*, 2013 ▸), which is capable of reading most of the common detector file formats. For array processing, *NumPy* (Oliphant, 2006 ▸; https://www.numpy.org) is used. The fitting is performed using *lmfit* (Newville *et al.*, 2014 ▸).

## 4. Documentation and availability   

The program is released under the terms of the GNU General Public Licence, either version 3 of the licence or any later version, and is available at https://owncloud.gwdg.de/index.php/s/JGluHlEp0pEUcAH. We only request that, for any published use, reference is made to the present publication. A user manual and test data are also provided at this address.

## Supplementary Material

User manual. DOI: 10.1107/S1600576719012159/vh5104sup1.pdf

## References

[bb1] Hunter, J. D. (2007). *Comput. Sci. Eng.* **9**, 99–104.

[bb2] Knudsen, E. B., Sørensen, H. O., Wright, J. P., Goret, G. & Kieffer, J. (2013). *J. Appl. Cryst.* **46**, 537–539.

[bb3] Kraus, W. & Nolze, G. (1996). *J. Appl. Cryst.* **29**, 301–303.

[bb4] Lundh, F. (2005). *An Introduction to Tkinter*, http://effbot.org/tkinterbook/.

[bb5] Macrae, C. F., Bruno, I. J., Chisholm, J. A., Edgington, P. R., McCabe, P., Pidcock, E., Rodriguez-Monge, L., Taylor, R., van de Streek, J. & Wood, P. A. (2008). *J. Appl. Cryst.* **41**, 466–470.

[bb7] Neher, S. H., Klein, H. & Kuhs, W. F. (2018). *J. Appl. Cryst.* **51**, 1352–1371. 10.1107/S1600576719012159 PMC6878879 31798363

[bb8] Newville, M., Stensitzki, T., Allen, D. B. & Ingargiola, A. (2014). *LMFIT: Non-Linear Least-Square Minimization and Curve-Fitting for Python*, https://doi.org/doi:10.5281/zenodo.11813.

[bb9] Oliphant, T. E. (2006). *A Guide to NumPy.* Trelgol Publishing.

