# Supplementary material for: FXD-CSD-GUI: a graphical user interface for the X-ray-diffraction-based determination of crystallite size distributions
Source: J Appl Crystallogr. 2019 Oct 22;52(Pt 6):1437–9. doi: 10.1107/S1600576719012159 (PMC6878879; doi:10.1107/S1600576719012159)
Supplement: Supplementary file 1 [file j-52-01437-sup1.pdf]

2019

FXD-CSD-GUI

USERS MANUAL V.1.0

DR. S. H. NEHER, DR. PD. H. KLEIN AND PROF. DR W. F. KUHS

PUBLISHED IN THE JOURNAL OF APPLIED CRYSTALLOGRAPHY 52,
HTTPS://DOI.ORG/10.1107/S1600576719012159

Manual: FXD-CSD-GUI

Inhalt

1. Preparations and Requirements ... 2
  1.1 Technical requirements for FXD-CSD measurements ... 2
  1.2 Reference material and Crystallographic Information ... 2
  1.3 Supported Image formats ... 2
2. Installation ... 2
  2.1 Python installation ... 3
  2.2 Installing python packages ... 3
  2.3 Install FXD-CSD-GUI ... 3
3. FXD-CSD-GUI ... 5
  3.1 Run the GUI ... 5
  3.2 Initiating an Analysis ... 6
    3.2.1 Choosing images ... 6
    3.2.2 *hkl* ring picking ... 6
  3.3 Complementing and testing the *hkl* settings ... 8
  3.4 Running *PickPeaks* ... 10
  3.5 Running *IntExtract* ... 10
  3.6 Conditioning the obtained data ... 11
  3.7 S1 Calculation ... 12
    3.7.1 Loading reference CSD data ... 12
    3.7.2 Using fitting parameter ... 12
  3.8 Results ... 13
4. References ... 14

FXD-CSD-GUI is a graphical user interface to the Python module *fxdcsd*. *fxdcsd* is written in Python 2.7, for crystal size distribution analysis using the fast X-ray diffraction crystal size distribution analysis method (FXD-CSD). The Program is published in (Neher *et al.*, 2019). The method is described in (Neher *et al.*, 2018).

1. Preparations and Requirements

1.1 Technical requirements for FXD-CSD measurements

To carry out FXD-CSD measurements a diffractometer with at least one precise rotation axis and a digital area detector is needed. It is required to mount the sample on the diffractometer head and perform stepwise rotation scans in transmission geometry. Each step has to result in one single frame and all its measurement parameters need to be available (e.g. stored in header). The samples have to be rotated for several degrees while series of frames are taken. The datasets given as examples here (see https://owncloud.gwdg.de/index.php/s/JGluHlEp0pEUcAH) are measured with a Bruker APEX II CCD diffractometer on a D8 Base. The samples are rotated for 10° around the ϕ-axis with 0.025° steps. More details on measurement strategies are given in (Neher *et al.*, 2018).

1.2 Reference material and Crystallographic Information

To perform a FXD-CSD analysis a *reference* material is mandatory. The *reference* material has to be a single crystalline powder with a crystallite size distribution (CSD) located in the lower micrometer range. The CSD hast to be measured with a different method (e.g. scanning electron microscopy or light scattering methods) and is ideally available as individual crystallite volume data entries (example available online). If the CSD is not available ins such format one may proceed with mean value and spread of the CSD. Furthermore, the crystallographic structure information of *reference* and *sample* need to be known. For more details see (Neher *et al.*, 2018).

1.3 Supported Image formats

The FXD-CSD-GUI is using the python module *fabIO* (Knudsen *et al.*, 2013) to read in the detector frames, which natively supports several file formats (e.g. Bruker sfrm files, Pilatus tif files, Mar mccd files and standard tif files). For more information on this topic please see (Knudsen *et al.*, 2013).

2. Installation

To use the FXD-CSD-GUI and the *fxdcsd* Python package, Python 2.7.X and several additional Python packages are needed. In the following sections the installation steps are described.

2.1 Python installation

Windows 7/10: Download the newest Python 2.7.x version for your Windows version form www.python.org and install it. If possible, use the option “Install for all users”, using the default directory “C:\Python27”. If you change the directory, note it down somewhere. The Directory path is needed during the setup.

After the Python installation, the system PATH variable needs to be set. To do this:

1. Open the Windows Explorer (press ⊞+E), right click on “Computer” and choose “Properties”
2. In the now open window click the “Advanced system settings” link. One needs administrator rights to be able to do this!
3. In the active tab click on “Environment Variables”.
4. In the lower menu choose the “Path” variable and press “Edit”. Now specify the value of the **PATH** environment variable by adding (trailing) the two paths “; C:\Python27; C:\Python27\Scripts”. Each entry is separated by a semicolon. Confirm by pressing twice the OK button.
   Windows 10: In Windows 10 the different path variable entries are arranged as list. Add the two entries on by one.
5. Open a command prompt (e.g. by searching for “cmd.exe” in the Windows start menu) and type in “pip” and hit enter. This should start the python package-management system. If an error massage is returned check whether step 1 to 4 was carried out correctly.
6. Start the python interpreter using the command prompt by typing in “python” and hit enter. If this does not work, check whether step 1 to 4 are carried out correctly. If the interpreter is running check whether it is using the correct Python version. The version number shown in the command prompt window must be the same as the one you just downloaded and installed.

Linux: We highly recommend using a 3rd party Python distribution like Anaconda or Canopy (Enthought). Personally, we have made good experience with Canopy (on Linux/Ubunt and Windows).

2.2 Installing python packages

Windows 7/10: To use FXD-CSD-GUI several python packages need to be installed. This is done by using the python package-management system pip.

1. Open a command prompt window
2. Type “pip install *numpy*” and hit enter. This should install the *numpy* package.
3. Repeat step 3 for *matplotlib*, *futures*, *lmfit*, *FabIO and scikit-image*

Linux: The needed Python packages (*matplotlib*, *futures*, *lmfit*, *FabIO and scikits-image*) have to be installed using the included package manager. For further information see the distribution manual.

2.3 Install FXD-CSD-GUI

Windows: Download *fxdcsd* from https://owncloud.gwdg.de/index.php/s/JGluHlEp0pEUcAH and unzip the file. Unless the installation path was not changed, copy the folder “fxdcsd” to

“C:\Python27\Lib\site-packages”. To run the GUI the user needs to execute the file “FXD-CSD-GUI.py”. This can be done at any location, therefore the file can be copied wherever desired.

Linux: Download *fxdcsd* from (see above) and unzip the file. Copy the folder fxdcsd to the location for 3rd party package “..\Lib\site-packages”.

3. FXD-CSD-GUI

3.1 Run the GUI

Execute the *FXD-CSD-GUI.py* via the command prompt line in window or in the terminal (linux). If not already done, open a command prompt (Windows start menu – search “cmd.exe”) and change the directory to the correct location (currently located at the Desktop). In the correct loaction type “python *FXD-CSD-GUI.py*” and press enter (the first start is likely to take some time).

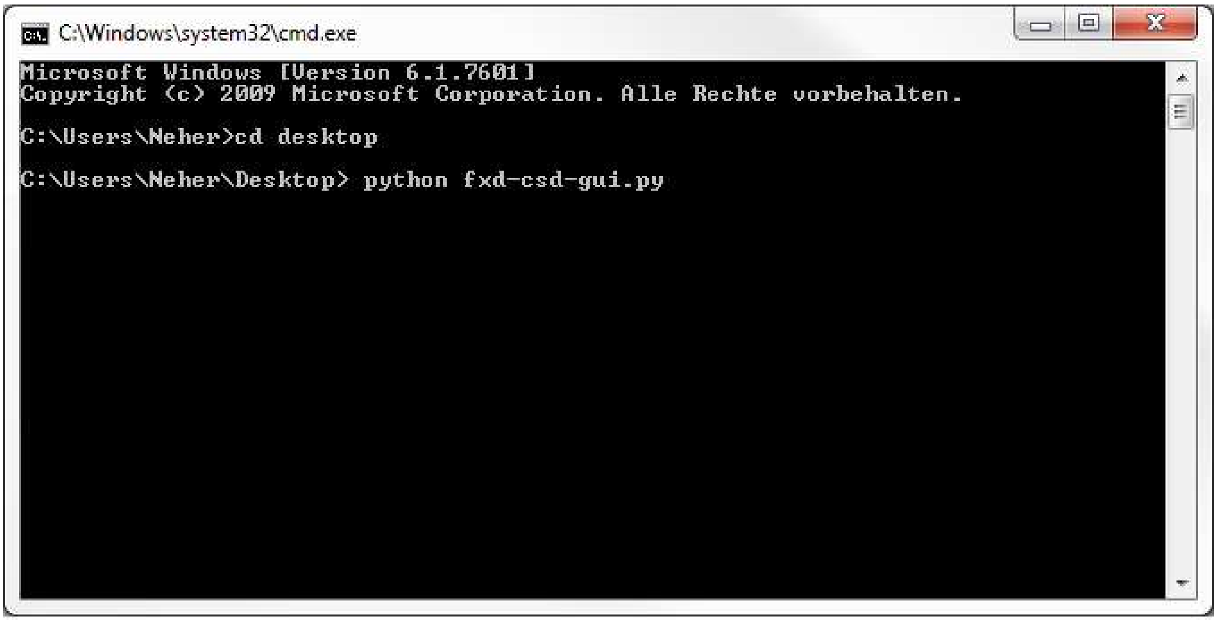

Figure 1: Windows command prompt. 1st input line “`cd desktop`” is changing the directory to the desktop. The 2nd input “`python fxd-csd-gui.py`” is starting the program.

3.2 Initiating an Analysis

This step is initiating the FXD-CSD analysis and must be carried out two times, once for the *reference* and once for the sample dataset. In the program interface and in the following description the *reference* is abbreviated STD and the *sample* as SAMP. All options and actions concerning the STD are highlighted in blue. The program window area concerning the SAMP is highlighted in orange (Figure 2).

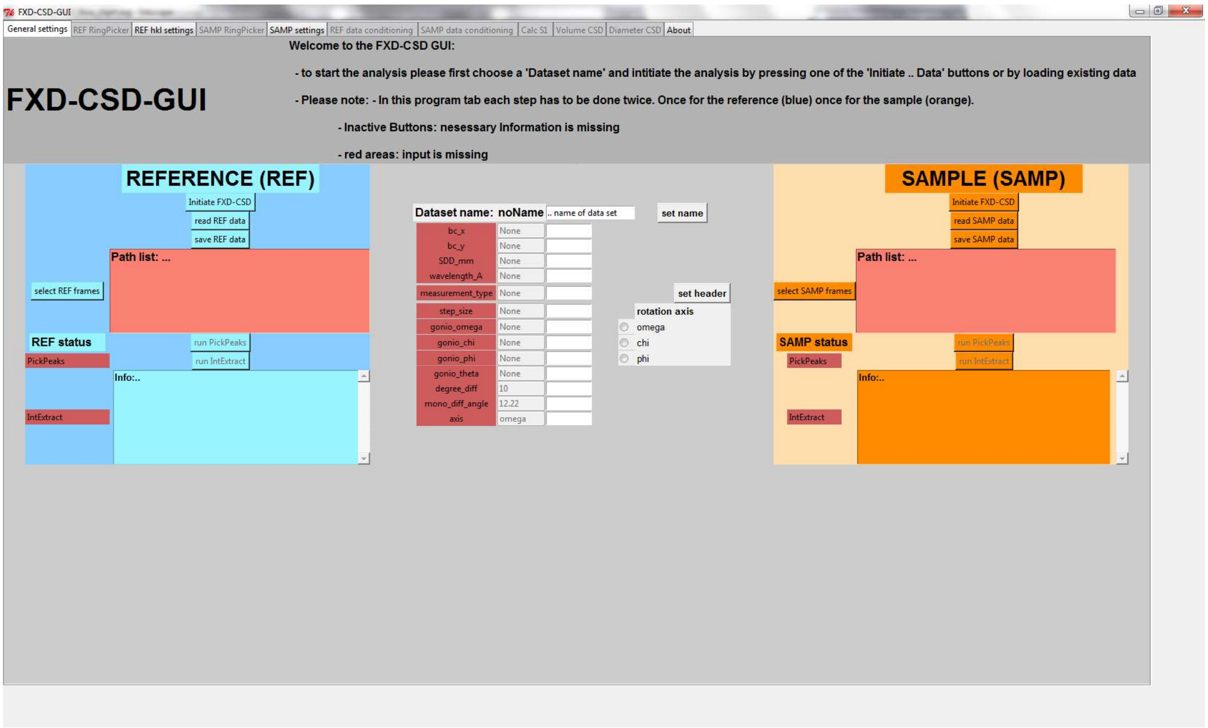

Figure 2: FXD-CSD-GUI with program tab "Settings general" active. Areas highlighted in red are missing important information. They turn green when a correct input has been made. Most program tabs are inactive because important information is missing.

3.2.1 Choosing images

- Pres one of the “Initiate FXD-CSD” buttons (STD or SAMP) and select all Images you want to analyze (use Ctrl + Shift to select several files at once). It does not matter whether one starts with the STD or SAMP data. One of course has to choose the according images. Confirm the selection by pressing the “Open” button.
- Depending whether the STD or SAMP images are selected the “STD *RingPicker*” or the “SAMP *RingPicker*” program tab is now active (Figure 3). On the left side the tab is showing an average frame which is computed from the loaded frames. The average is used to give a better impression of the ring locations. On the right side of this tab, the frame contrast can be altered and the *hkl*s can be provided.

3.2.2 *hkl* ring picking

For this step a diffractogram of the analyzed material should be at hand. The *hkl*s and their *2Θ* values have to be known.

- To start the interactive ring picking, type in a *hkl* in to the entry field named “provide *hkl*”. *Hkl*s have to be provided as plain numbers and “-“ signs to mark negative indices. The input is confirmed by pressing the “new ring” button. **Example:** The {002} for example has to be typed in as “002” and the $\{11\bar{2}\}$ as “11-2”.
- Now the according *hkl* ring can be defined by picking single spots with the left mouse button on that ring. As soon as four spots are marked a circle will appear on the screen. The circle plots a first solution, connecting the already marked positions. One can add more positions until the desired *hkl* ring is sufficiently traced by the red circle.
- If a marker is accidentally misplaced it can be deleted by pressing the “remove last point” button. By pressing the “del Ring” button the complete ring is deleted (Figure 4).
- If more than one *hkl* ring is marked, it is possible to switch between them by using the radio buttons, located below the “provide *hkl*” entry field. The active *hkl* ring is marked red and can be modified or deleted (Figure 4).

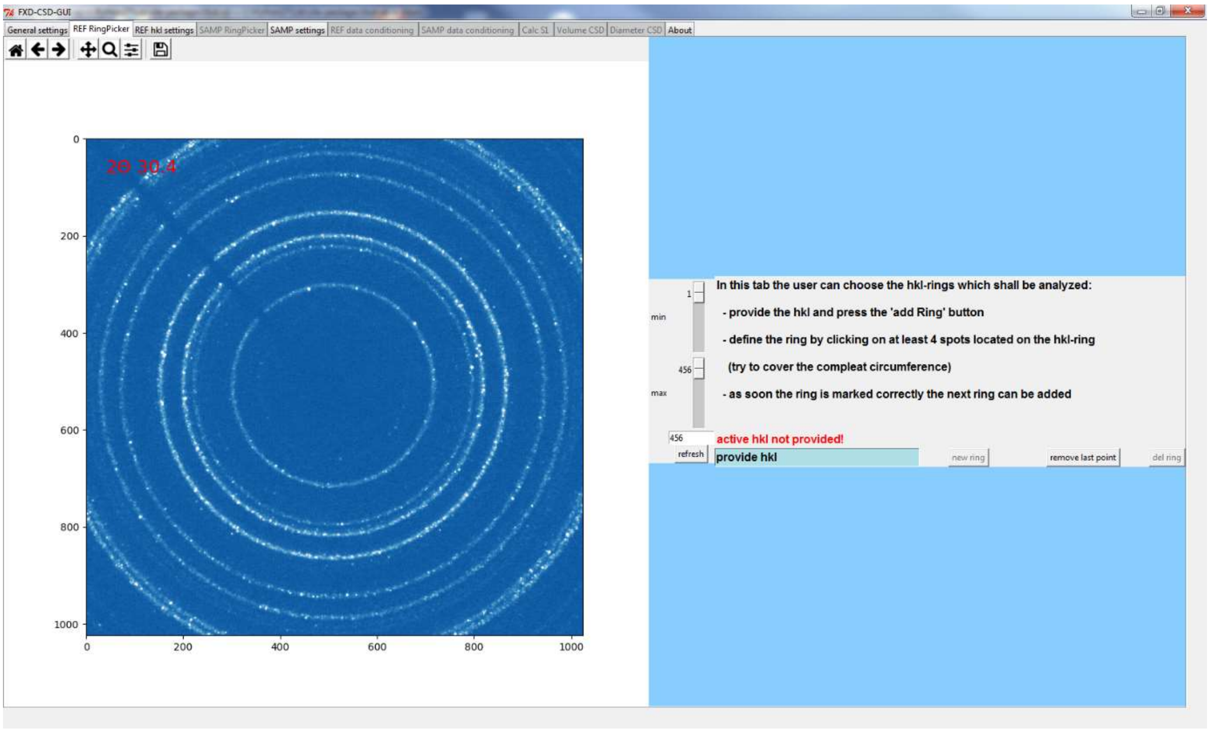

Figure 3: Active “STD *RingPicker*” program tab. This tab is showing an averaged spotty diffraction pattern (left side) and lets the user add *hkl*rings by typing in *hkl* indices (right side). In the upper left corner, there is the standard toolbar which lets the user manipulate the image plot and allows the user to save the figure.

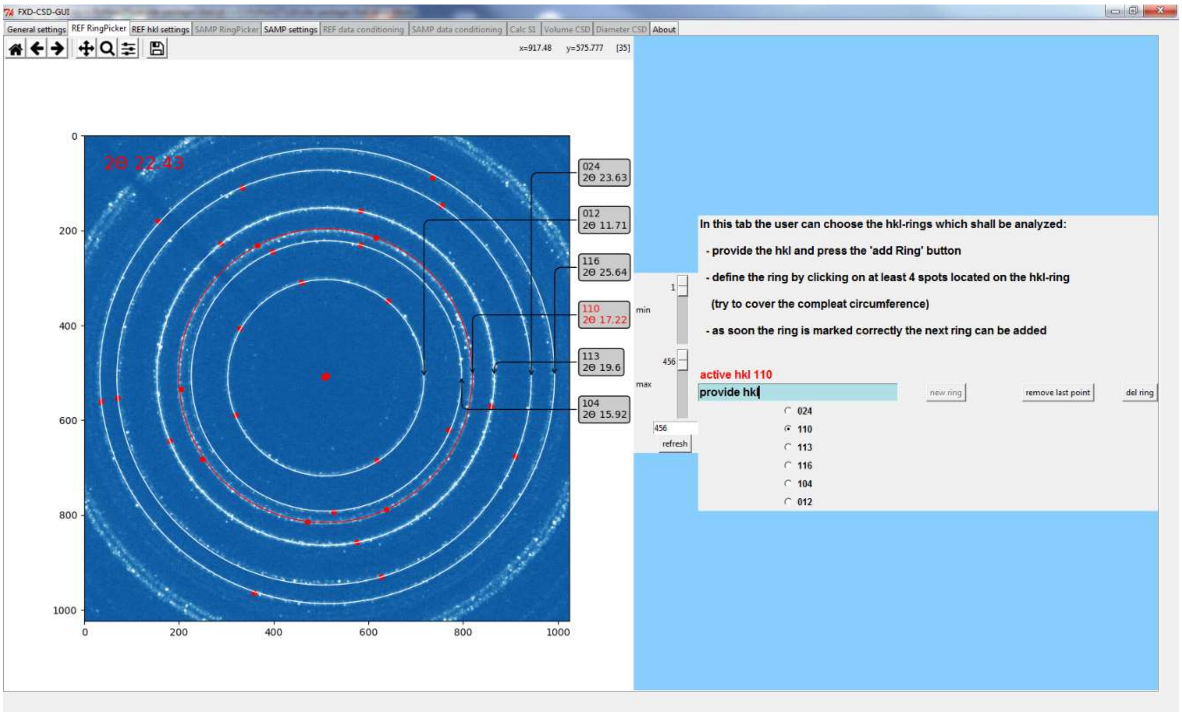

Figure 4: Active "STD *RingPicker*" program tab. Six *hkl* rings are already selected by 1) Typing in the *hkl* into the entry field and 2) selecting at least 4 peaks on the correct *hkl* ring by clicking on peaks with the left mouse button. The active ring is shown in red. The radio buttons below the entry field allow the user to switch between the entered *hkl* rings. The upper left corner shows the standard toolbar which lets the user manipulate the image plot and allows the user to save the figure.

3.3 Complementing and testing the *hkl* settings

For both the STD and SAMP a program tab, named "STD *hkl* settings" or "SAMP *hkl* settings" exists. In this tab all material and measurement specific settings can be altered (Figure 5). These are either measurement (e.g. the exposure time) or *hkl* specific (e.g. the ring radius or ring width). Again, red colored labels show that information is missing.

- To provide missing information or to change already existing information fill in the entry field and press "set header".
- The radio buttons labeled "mult" and "abs" are used to change the threshold type. When "abs" is selected an absolute and user provided threshold value is used. When "mult" is chosen instead the user provided value is used to multiply the background level which is applied as threshold. The radio buttons "in" and "out" define where the background level is determined; an inner or outer adjacent rind shape area. The background area position can only be changed when the type "mult" is chosen. (Figure 6 and 7).
- By pressing the "test *PickPeaks* settings" button the provided information is used to test the *PickPeaks* function with a single frame. The result is shown in two plots (Figure 6 and 7).
- With the "test *PickPeaks* settings" testing tool it is possible to change all parameters. Especially the threshold and the ring widths have to be tuned precisely to achieve a precise intensity extraction. Figure 6 and 7 for example show the results with three *hkl* rings ({113},{024} and {116}). The 113 *hkl* ring is using an absolute threshold. The other two *hkl* rings are set to use a threshold multiplier obtained from an adjacent inner background area. This value is modified by the "threshold" value, provided for each ring and used as multiplier.
- Once all settings are successfully tuned the final *PickPeaks* analysis can be started.

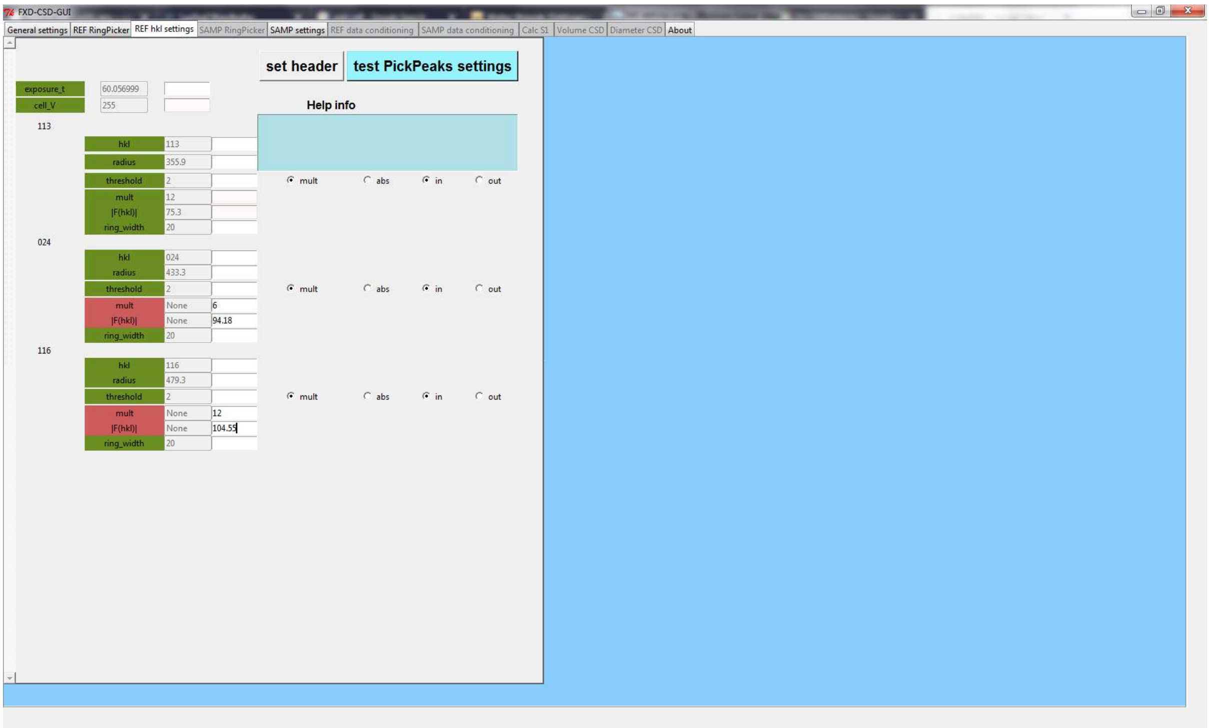

Figure 5: Active “STD *hkl* settings” programm tab. Three *hkl* rings are listed, two of them are in the state of editing (left side, read fields). The given entries are ready to be set by pressing the “set header” button. The threshold type and position can be set with the radiobuttons (right site). To test the settings, the “test *PickPeaks* settings” button can be prest to run the *PickPeaks* function on one singel frame.

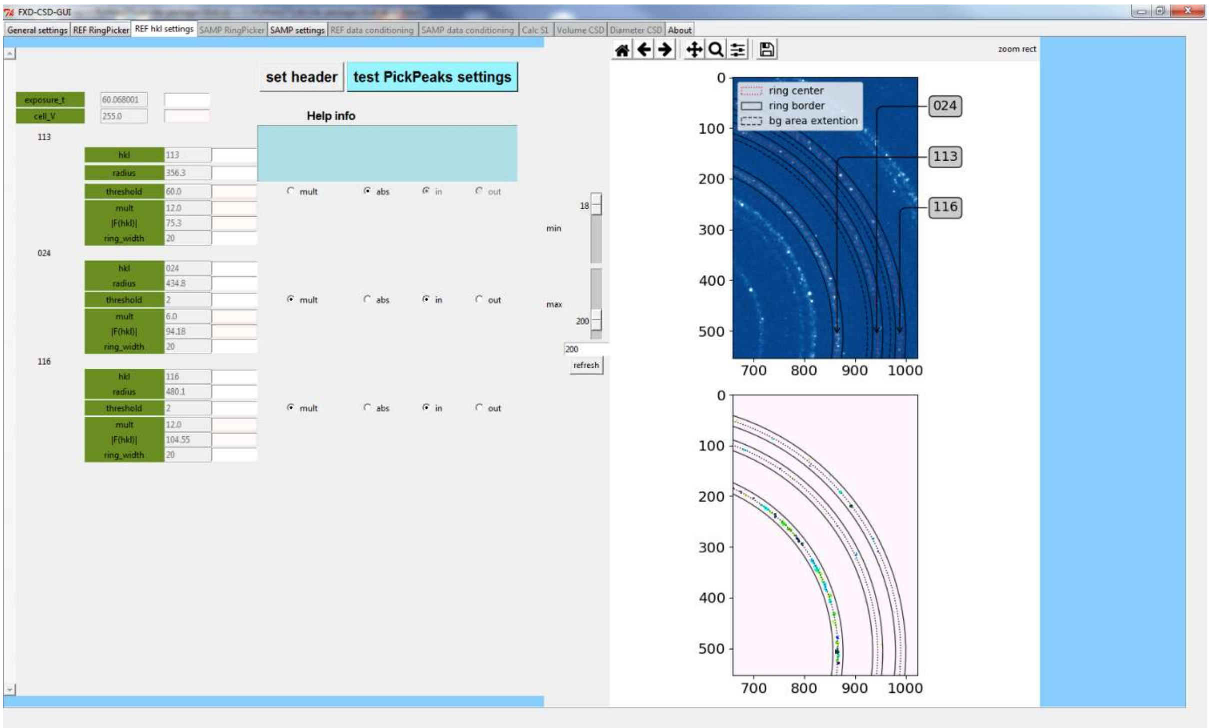

Figure 6: Active “STD *hkl* settings” programm tab. Three *hkl* rings are listed (left side) and the result of running the test PickPeaks function is shown on the left side (compare Figure 7). The two plots are zoomed in using the toolbar.

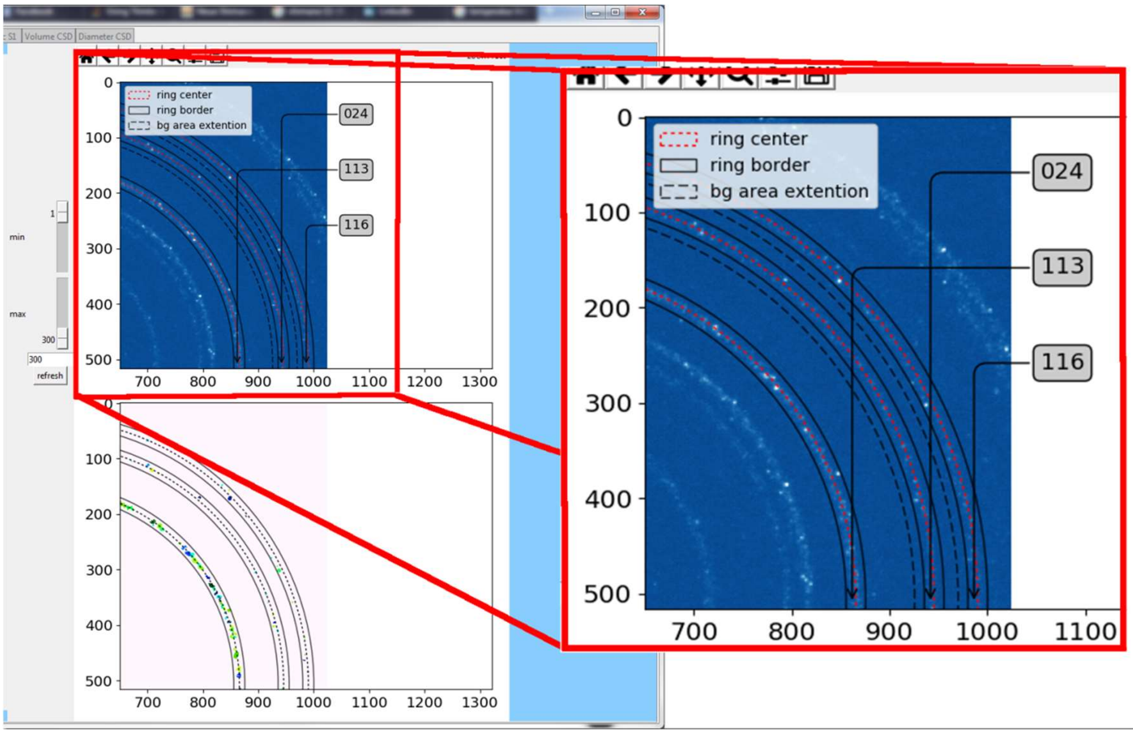

Figure 7: Section of the active “STD *hkl* settings” programm tab with the two plotts in focus (compare figure 6). The plots are zommed in on one quarter of the hkl rings and one is enlarged in the inset. The upper of the two plots shows the three rings or area of interst (definded by ring radius and ring width) of each *hkl* ring. In case of the two outer rings ({024} and {116}), the boundry line of the area use for the background determination is shown as well. The inner most ring ({113}) is using an absolute value for the threshold determination (compare Figure 6).

3.4 Running *PickPeaks*

The *PickPeaks* function can be run by pressing the “run *PickPeaks*” button on the “General settings” program tab (Figure 2) and is detecting the individual intensity peaks on the *hkl* rings. The function has to be started separately for the STD and the SAMP data. Once the function is started all loaded frames are processed one by one. The progress is shown in the info window and in the command line prompt. The time needed for processing does highly depend on whether an absolute threshold is used, or a threshold area is defined. Using an absolute value is much faster. Furthermore, is the number of frames, the frame size and the computing power available, of great importance for the computation time. It can take several hours on a standard PC.

After all images are analyzed the information is merged. The end of the merging is shown by the message “3D Peak merging … ready!“ within the info window.

3.5 Running *IntExtract*

As soon as the *PickPeaks* process is done the *IntExtract* function can be run by pressing the “run *IntExtract*” button (Figure 2). This again can take several hours depending on the number of peaks detected and the computing power of the computer in use. In this step the individual peak intensities are extracted and turned into intensity rocking curves via intensity integrations in the two directions within the image plane.

3.6 Conditioning the obtained data

Once all datasets are analyzed, the results can be examined in the data conditioning tabs – there is one for the STD and one for the SAMP (Figure 8). Four plots are showing the intensity distribution for all analyzed and selected *hkl* rings in different stages of data corrections (Neher *et al.*, 2018). On the left side the used *hkl* rings can be chosen "used *hkl*". One *hkl* ring hast to be dedicated for internal intensity scaling "int. scaling". By pressing the "run data conditioning" button the plots are recalculated for the current selection and settings. By pressing the "export data as .csv" the selected data is stored to disk.

If three or more *hkl* rings are measured the shown plots facilitate data quality evaluation. If measured correctly, the intensity distributions in the two lower plots (after all corrections and intensity normalization) should match in shape and position (Neher *et al.* 2018). A possible error sources is a CSD partially below or near the lower detection limit and is called $ID_{cut\text{-}off}$ (on the left side distribution tail). $ID_{cut\text{-}off}$ is describing the effect of the structure factor variations between the different *hkl*-planes on the shape of the measured *hkl* intensity distribution. A second possible error source is called $ID_{exaggeration}$ (right side distribution tail) which is originating from different degrees of peak overlap due to overcrowded ring occupancy depending on multiplicity and ring radius (Neher *et al.* 2018).

To be able to evaluate the results in such way, the user needs to take several factors in to account. These are: 1) the multiplicity, which is influencing the ring occupancy ($ID_{exaggeration}$), 2) the ring radius which is influencing the angular resolution ($ID_{exaggeration}$) and 3) the structure factor which in case of a CSD near the lower detection limit can lead to $ID_{cut\text{-}off}$ appearing for *hkl* rings with smaller structure factors.

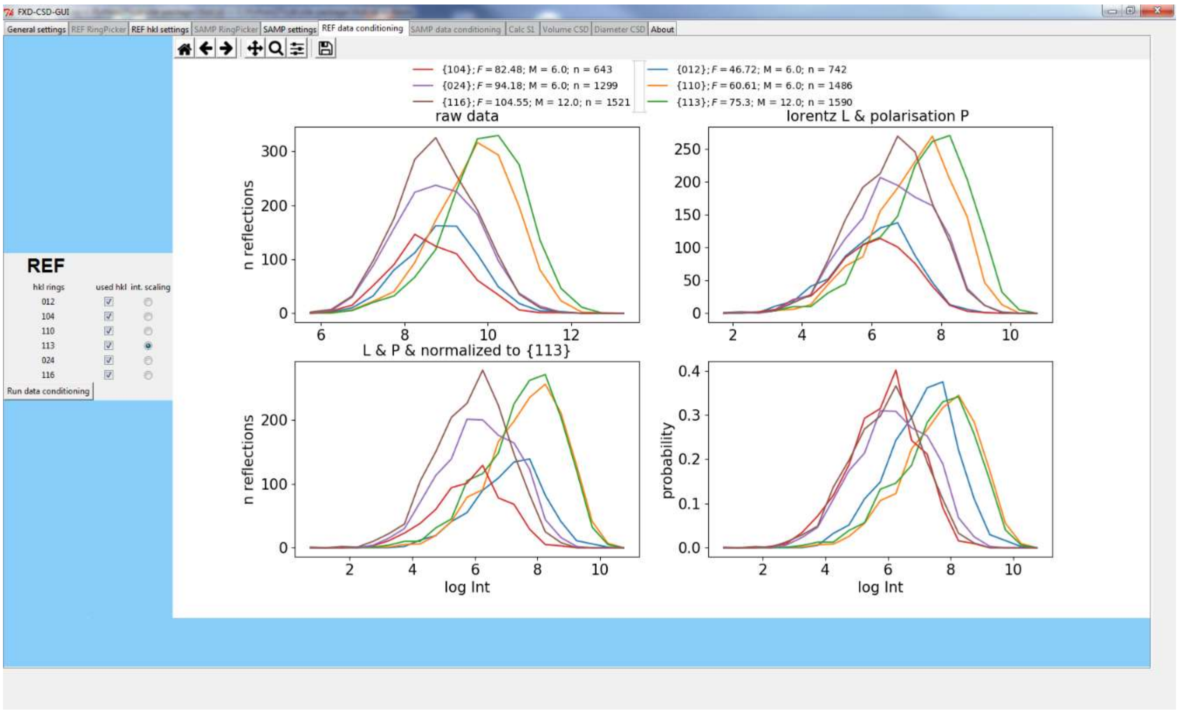

Figure 8: Active "STD data conditioning" program tab. The four plots on the right side show the selected intesity distributions in different data correction and scaling stages. On the left the user is able to change the selection and recalculate the plots. Only the chosen and shown intensity distributions are used for the later on. The data shows, that the displayed intensity distributions do not match in position after the corrections and scaling is applied. In this case it is most

likely that the {113}, {110} and the {012} are too crowded (compare Figure 4). Therefore, for the further analysis one should deselect the {113}, {110} and the {012} *hkl* rings.

3.7 S1 Calculation

As soon as step 3.6 is carried out for STD and SAMP the “Calc 1” program tab is activated (Figure 9; at first there will be no plot visible). In this tab the user is able to control the S1 scaling factor determination between the measured intensity distribution (summation of all selected STD IDs) and the volume based CSD information data (see 3.7.1) or CSD fitting parameters (see 3.7.2). It is also possible to change the histogram bin size by changing the entry values.

3.7.1 Loading reference CSD data

Push the “Open reference csv file” to browse the file location and choose the desired csv file. The data has to be provided as single column; comma separated text file. Each data entry in the file carries the volume information of a single grain in nm^3. An example file is provider in the downloaded ‘fxdcsd’ folder.

3.7.2 Using fitting parameter

If no reference volume CSD data file is available, the analysis can be done with distribution fitting parameters. To do so, alter the STD CSD mean and STD CSD sigma value (both as ln(Vol)[nm^3]) in the entry fields and press “Determine S1 and SAMP CSD” button. On the right side the fitting parameters are shown. The user can choose whether to use a Gaussian distribution, a log-normal distribution or a skewed-Gaussian distribution function. Furthermore, it is possible to use a custom fitting model. To do this one need to activate the according button and define the function in the “custom_model.py” file located in the “fxdcsd” module folder. The defined function is imported into the program and used by the *lmfit* fitting module. A Gaussian distribution is given as an example. Further information is given in the *lmfit* (Newville *et al.*, 2014) module online documentation.

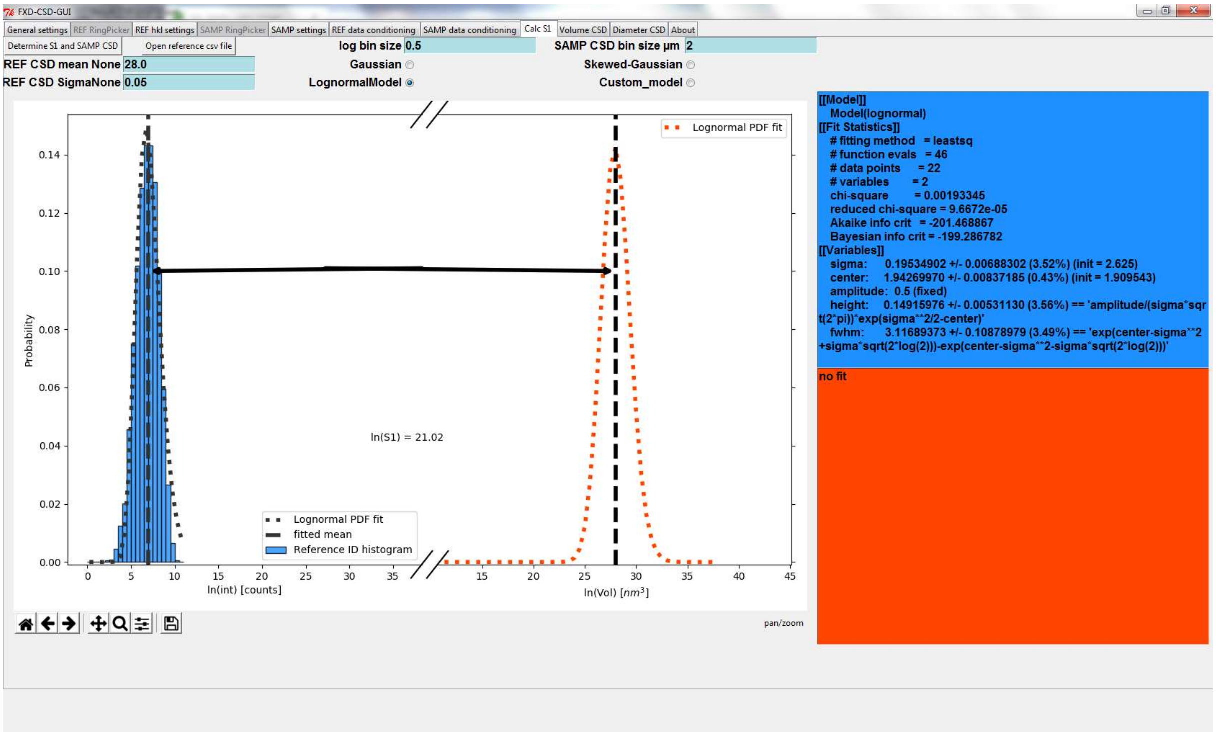

Figure 9: Active “Calc S1” program tab with S1 determination plot. The left histogram shows the measured intensity distribution with a log-normal function fitted to the data. The right part of the plot is showing the log-normal distribution calculated from the provided mean and sigma values.

3.8 Results

With pressing the “Determine S1 and SAMP CSD” button the program tabs “Volume CSD” and “Diameter CSD” (Figure 10) turn active and show the final analysis results. On the left the CSD is shown as histogram. On the right the fitting parameters and the histogram data is shown. The shown data points are the bin midpoints and the probability and can be copied for further usage. Different distribution function can be tested in the same way as described for the S1 determination.

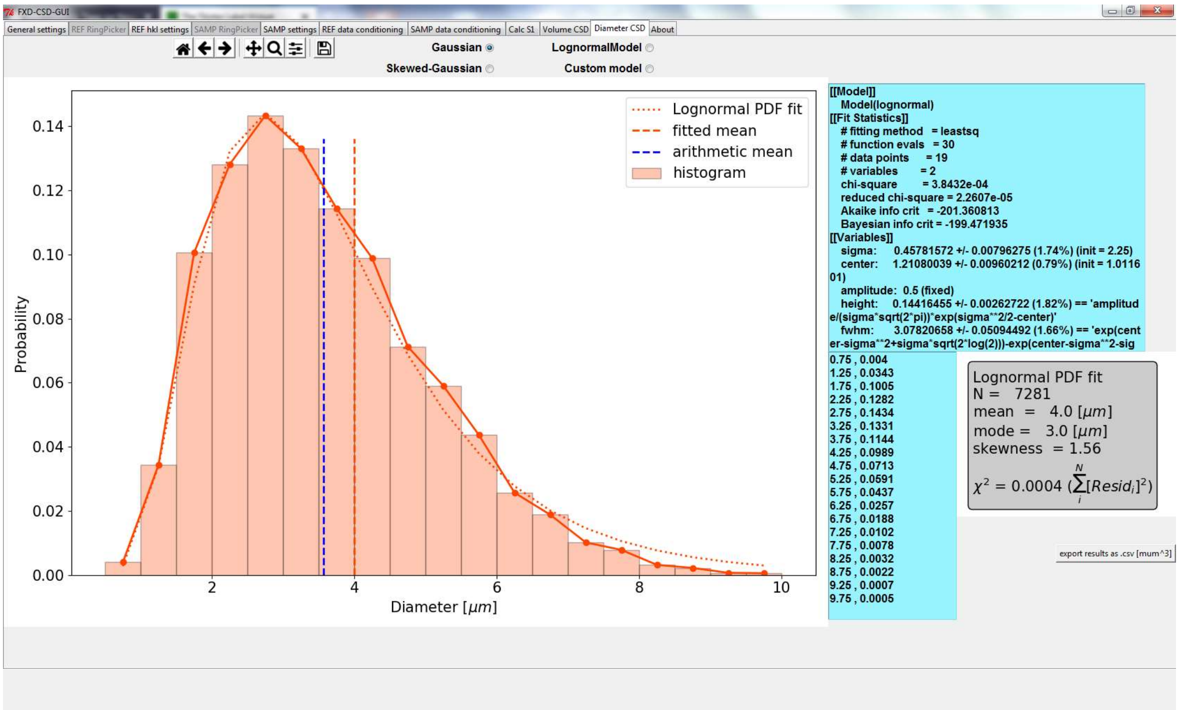

Figure 10: Active "Diameter CSD" program tab. The plot shows the CSD histogram including a log-normal probability density function fitted to the data. The fitting parameters are shown in the gray text field.

4. References

Knudsen, E. B., Sørensen, H. O., Wright, J. P., Goret, G. & Kieffer, J. (2013). *J. Appl. Crystallogr.* **46**, 537–539.

Neher, S. H., Klein, H. & Kuhs, W. F. (2018). *J. Appl. Crystallogr.* **51**, 1352–1371.

Neher, S. H., Klein, H. & Kuhs, W. F. (2019). *J. Appl. Crystallogr.* **52**,.

Newville, M., Stensitzki, T., Allen, D. B., Rawlik, M., Ingargiola, A. & Nelson, A. LMFIT:Non-Linear Least-Square Minimization and Curve-Fitting for Python, (2014). doi:10.5281/zenodo.11813.
